# Developmental stages of peach, plum, and apple fruit influence development and fecundity of *Grapholita molesta* (Lepidoptera: Tortricidae)

**DOI:** 10.1038/s41598-021-81651-4

**Published:** 2021-01-22

**Authors:** Souvic Sarker, Young Ha Woo, Un Taek Lim

**Affiliations:** Department of Plant Medicals, Andong National University, Andong, 36729 Republic of Korea

**Keywords:** Ecology, Population dynamics

## Abstract

Host plant attributes are essential factors determining the population dynamics of herbivorous insects. The developmental stage of host plants, in particular, may affect the biology of *Grapholita molesta* (Busck), a possibility that has rarely been examined. Here we assessed the effect of developmental stage of plum, peach, and apple fruits on the development and fecundity performance of *G. molesta,* along with an examination of the firmness and sugar content of the fruits. Among the fruits collected earliest (May 31), plum and apple were better food sources for *G. molesta* compared to peach in terms of development, reproduction, and life table parameters. However, despite the higher sugar content in peach, *G. molesta* larvae showed a lower rate of fruit penetration in peach, probably due to fruit firmness. In the later-collected fruit (June 25), both peach and apple were better than plum, as peach and apple were softer and had higher sugar content. Nevertheless, the penetration rate of larva was still low in peach probably due to pubescence on the fruit surface. Although the plum fruits in the later collection date were softer with higher sugar content, there was a negative impact on the development and reproduction because fruits started to liquefy earlier. In conclusion, the developmental stage of fruits with changes in fruit firmness or sugar content affected the development and reproduction of *G. molesta,* and apple would be the best food source.

## Introduction

The oriental fruit moth, *Grapholita molesta* (Busck) (Lepidoptera: Tortricidae), is a globally important insect pest^1^. It is widespread across temperate and subtropical regions in Asia, Europe, the Americas, Africa, and Australia^2,3^, with a host range encompassing species in the Rosaceae, including most species of *Prunus* and *Pyrus*^2^. The stone fruits, plum *Prunus domestica* L. and peach *Prunus persica* (L.) (Rosales: Rosaceae), are considered the primary hosts of *G. molesta*, whereas the pome fruits, pear *Pyrus communis* L. (Rosales: Rosaceae) and apple *Malus domestica* Borkh. (Rosales: Rosaceae), are considered secondary hosts^2,4,5^. The pest undergoes 3–6 generations per year depending on the location and temperature^6–10^.

Many invasive herbivores have a common characteristic of utilizing a broad range of host plants^11,12^. The population dynamics of herbivorous insects depend on the availability of suitable host plants^13,14^, and the phenology of host plants in turn influences the performance (such as development time, survival rate, and fecundity) of herbivorous insects^15–17^. Previous studies have showed that the phenology of host plants influences the seasonal dynamics of the stink bug *Halyomorpha halys* (Stål)^18^ and the survival or fitness of the tortricid moths *Choristoneura rosaceana* (Harris)^19^ and *Choristoneura fumiferana* (Clemens)^20^. Larvae of *G. molesta* feed at different sites on peaches and apples over the course of the growing season^21^, and Du et al.^1^ showed how phenological succession of peach and pear plants can influence the population dynamics of *G. molesta.*

The larval survival of *G. molesta* is better on shoots than on fruits in peach early in the season^1,22^, and thus *G. molesta* larvae infest shoots more frequently early in the growing season. Later in the growing season, *G. molesta* moths are attracted more to fruits than twigs^23,24^. The firmness of the fruit in the early season may influence larval survival^17^, while sugar content may also affect larval development^25^. Our previous study^26^ found that immature plum or apple fruits were better food sources than peach, although larval survival was found to be better in ripening peach fruits than in immature ones^22^. However, the underlying causes of those discrepancies are still unknown. We hypothesize that firmness and sugar content may affect the development and reproduction of *G. molesta*, and to test this, we investigated the effect of the developmental stages of plum, peach, and apple fruit on the development, reproduction, and life table parameters of *G. molesta,* while also examining fruit firmness and sugar content.

## Results

### Stage development and survival rates

Egg duration of *G*. *molesta* was not affected by fruit species (*F* = 0.54, *df* = 2, 261, *P* = 0.581), by collection date (*F* = 0.06, *df* = 2, 261, *P* = 0.941), or by fruit species by collection date interaction (*F* = 0.24, *df* = 4, 261, *P* = 0.914) (Table 1). Durations of other life stages were all significantly affected by fruit species (larva *F* = 28.16, *df* = 2, 144, *P* < 0.001; prepupa *F* = 4.38, *df* = 2, 133, *P* = 0.014; pupa *F* = 8.76, *df* = 2, 113, *P* < 0.001; larva-pupa *F* = 22.92, *df* = 2, 113, *P* < 0.001), by collection date (larva *F* = 17.43, *df* = 2, 144, *P* < 0.001; prepupa *F* = 7.73, *df* = 2, 133, *P* < 0.001; larva-pupa *F* = 11.59, *df* = 2, 113, *P* < 0.001) except pupa (*F* = 1.99, *df* = 2, 113, *P* = 0.142), or by fruit species by collection date interaction (larva *F* = 30.59, *df* = 4, 144, *P* < 0.001; prepupa *F* = 5.15, *df* = 4, 133, *P* < 0.001; pupa *F* = 8.41, *df* = 4, 113, *P* < 0.001; larva-pupa *F* = 16.12, *df* = 4, 113, *P* < 0.001).

**Table 1:** Durations (d ± SE) of each life stage of oriental fruit moth, *Grapholita molesta*, reared in plum, peach, and apple fruits collected at various times of the growing season.

Fruit	Collected on	Egg (d)	Larva (d)	Prepupa (d)	Pupa (d)	Larva- pupa (d)
Plum	May 11^ǂ^	3.13 ± 0.06	11.44 ± 0.61b^§^	3.47 ± 0.12b	7.67 ± 0.43b	23.13 ± 0.74b
	May 31	3.17 ± 0.07	10.79 ± 0.29bA^§§^	3.52 ± 0.18bC	9.11 ± 0.42bA	23.58 ± 0.49bA
	June 11	3.20 ± 0.07	14.93 ± 1.16aA	4.69 ± 0.33aA	10.18 ± 0.46aA	29.55 ± 1.03aA
	June 25	3.23 ± 0.08	13.93 ± 0.22aA	4.58 ± 0.15aA	10.80 ± 0.42aA	29.10 ± 0.46aA
Peach	May 31	3.30 ± 0.09	9.13 ± 0.40aB	4.25 ± 0.16aA	9.29 ± 1.25aA	22.57 ± 1.32aAB
	June 11	3.23 ± 0.08	8.82 ± 0.35aC	3.40 ± 0.22bB	9.44 ± 0.29aA	21.33 ± 0.38aC
	June 25	3.27 ± 0.08	8.58 ± 0.26aC	3.41 ± 0.15bB	8.56 ± 0.24aB	20.33 ± 0.33aB
Apple	May 31	3.20 ± 0.07	8.44 ± 0.22cB	3.13 ± 0.10bB	10.35 ± 0.33aA	21.74 ± 0.38cB
	June 11	3.27 ± 0.08	10.64 ± 0.49bB	3.86 ± 0.13aAB	10.24 ± 0.29aA	24.24 ± 0.60bB
	June 25	3.23 ± 0.08	12.68 ± 0.27aB	4.05 ± 0.20aAB	11.59 ± 0.56aA	28.06 ± 0.66aA

^ǂ^Excluded in two-way ANOVA.

^§^Means followed by different lower case letter are significantly different among collection dates within a fruit species.

^§§^Means followed by different upper case letter are significantly different among host fruits in each collection date.

Duration of the larval stage became longer in plum and apple as fruit collection date, but it showed opposite trend in peach. Among later-collected fruits (June 25), duration of the larval stage was shortest in peach.

Prepupal duration showed similar pattern as in larval duration. Pupal duration became longer as collection date in plum fruits, but no such trend was found in either peach or apple. In comparison on each collection date, no significance was found among fruits except June 25 when peach showed shortest pupal duration.

The total period from of larva plus pupa was also shortest when insects were reared on peach fruits collected on June 25 (20.33 d) compared to those reared on either plum or apple fruits collected on same date.

Hatching rate of eggs were not affected by fruit species (*F* = 3.44, *df* = 2, 4, *P* = 0.101), by collection date (*F* = 1.03, *df* = 2, 4, *P* = 0.413), or by fruit species by collection date interaction (*F* = 3.50, *df* = 4, 4, *P* = 0.126) (Table 2). The fruit penetration rate of first instars was not statistically different within each fruit developmental stages (*F* = 0.25, *df* = 2, 4, *P* = 0.789) or by fruit species by collection date interaction (*F* = 4.50, *df* = 4, 4, *P* = 0.087), but was significantly different between fruit species (*F* = 6.39, *df* = 2, 4, *P* = 0.033). For example, the fruit penetration rate was lowest in peach (0.40), compared to plum (0.87) or apple (0.83) collected on May 31 (*χ*^2^ = 19.37, *df* = 2, *P* = 0.009). The fruit exiting rate was also affected by fruit species (*F* = 5.35, *df* = 2, 4, *P* = 0.046) but not by collection date (*F* = 0.35, *df* = 2, 4, *P* = 0.720), or by fruit species by collection date interaction (*F* = 3.50, *df* = 4, 4, *P* = 0.126). The fruit exiting rate of mature larva was similar within each fruit species for all fruit collection dates. Among fruit species, the fruit exiting rate was highest in apple than peach on May 31 (*χ*^2^ = 10.63, *df* = 2, *P* = 0.005). Pupation rate and emergence rate were not affected by fruit species (pupation rate *F* = 1.37, *df* = 2, 4, *P* = 0.324; emergence rate *F* = 0.17, *df* = 2, 4, *P* = 0.848), by collection date (pupation rate *F* = 1.07, *df* = 2, 4, *P* = 0.401; emergence rate *F* = 1.04, *df* = 2, 4, *P* = 0.401), or by fruit species by collection date interaction (pupation rate *F* = 0.13, *df* = 4, 4, *P* = 0.966; emergence rate *F* = 0.15, *df* = 4, 4, *P* = 0.951). Total immature survival rate was affected by fruit species (*F* = 9.60, *df* = 2, 4, *P* = 0.014) or by fruit species by collection date interaction (*F* = 15.50, *df* = 4, 4, *P* = 0.011), but not by collection date (*F* = 0.15, *df* = 2, 4, *P* = 0.864). In general, the total immature survival rate was highest in apple collected on May 31 compared to peach (*χ*^2^ = 26.12, *df* = 2, *P* < 0.001).

**Table 2:** Survival rate of each life stage of oriental fruit, *Grapholita molesta*, moth reared in plum, peach, and apple fruits collected at various times of the growing season.

Fruit	Collected on	Hatching rate of egg	Fruit penetration rate of first instar larva	Fruit exiting rate of mature larva	Larval survival rate	Pupation rate	Emergence rate	Total immature survival rate
Plum	May 11^ǂ^	0.92 (619/675)	0.83 (25/30)a^§^	0.72 (18/25)a	0.60 (18/30)ab	0.94 (17/18)	0.88 (15/17)	0.50 (15/30)a
	May 31	0.93 (769/823)	0.87 (26/30)aA^§§^	0.92 (24/26)aAB	0.80 (24/30)aA	0.96 (23/24)	0.83 (19/23)	0.63 (19/30)aA
	June 11	0.94 (678/720)	0.80 (24/30)aA	0.63 (15/24)aB	0.50 (15/30)abAB	0.87 (13/15)	0.85 (11/13)	0.37 (11/30)aA
	June 25	0.94 (426/452)	0.73 (22/30)aAB	0.64 (14/22)aA	0.47 (14/30)bAB	0.86 (12/14)	0.83 (10/12)	0.33 (10/30)aA
Peach	May 31	0.95 (331/349)	0.40 (12/30)aB	0.67 (8/12)aB	0.27 (8/30)aB	1.00 (8/8)	0.88 (7/8)	0.23 (7/30)aB
	June 11	0.95 (765/808)	0.47 (14/30)aB	0.79 (11/14)aAB	0.37 (11/30)aB	0.91 (10/11)	0.90 (9/10)	0.30 (9/30)aA
	June 25	0.95 (984/1033)	0.50 (15/30)aB	0.80 (12/15)aA	0.40 (12/30)aB	1.00 (12/12)	0.75 (9/12)	0.30 (9/30)aA
Apple	May 31	0.93 (1274/1367)	0.83 (25/30)aA	1.00 (25/25)aA	0.83 (25/30)aA	0.92 (23/25)	1.00 (23/23)	0.77 (23/30)aA
	June 11	0.95 (814/861)	0.77 (23/30)aA	0.96 (22/23)aA	0.73 (22/30)aA	0.95 (21/22)	0.81 (17/21)	0.57 (17/30)aA
	June 25	0.95 (1337/1409)	0.80 (24/30)aA	0.92 (22/24)aA	0.73 (22/30)aA	0.91 (20/22)	0.85 (17/20)	0.57 (17/30)aA

^ǂ^Excluded in two-way ANOVA.

^§^Means followed by different lower case letter are significantly different among collection dates within a fruit species.

^§§^Means followed by different upper case letter are significantly different among host fruits in each collection date.

Within individual fruit species, larval survival rate was similar across collection dates (*F* = 0.25, *df* = 2, 4, *P* = 0.789), but there were significant differences among fruit species (*F* = 9.03, *df* = 2, 4, *P* = 0.015), or by fruit species by collection date interaction (*F* = 15.50, *df* = 4, 4, *P* = 0.011). For example, larval survival rate in apple was significantly higher than other fruits, and this was true for all developmental periods (0.83, 0.73, and 0.73) compared to peach (0.27, 0.37, and 0.40) (Table 2). No significant differences in larval survival rate were observed between apple and plum in all developmental stages.

**Figure 1:**
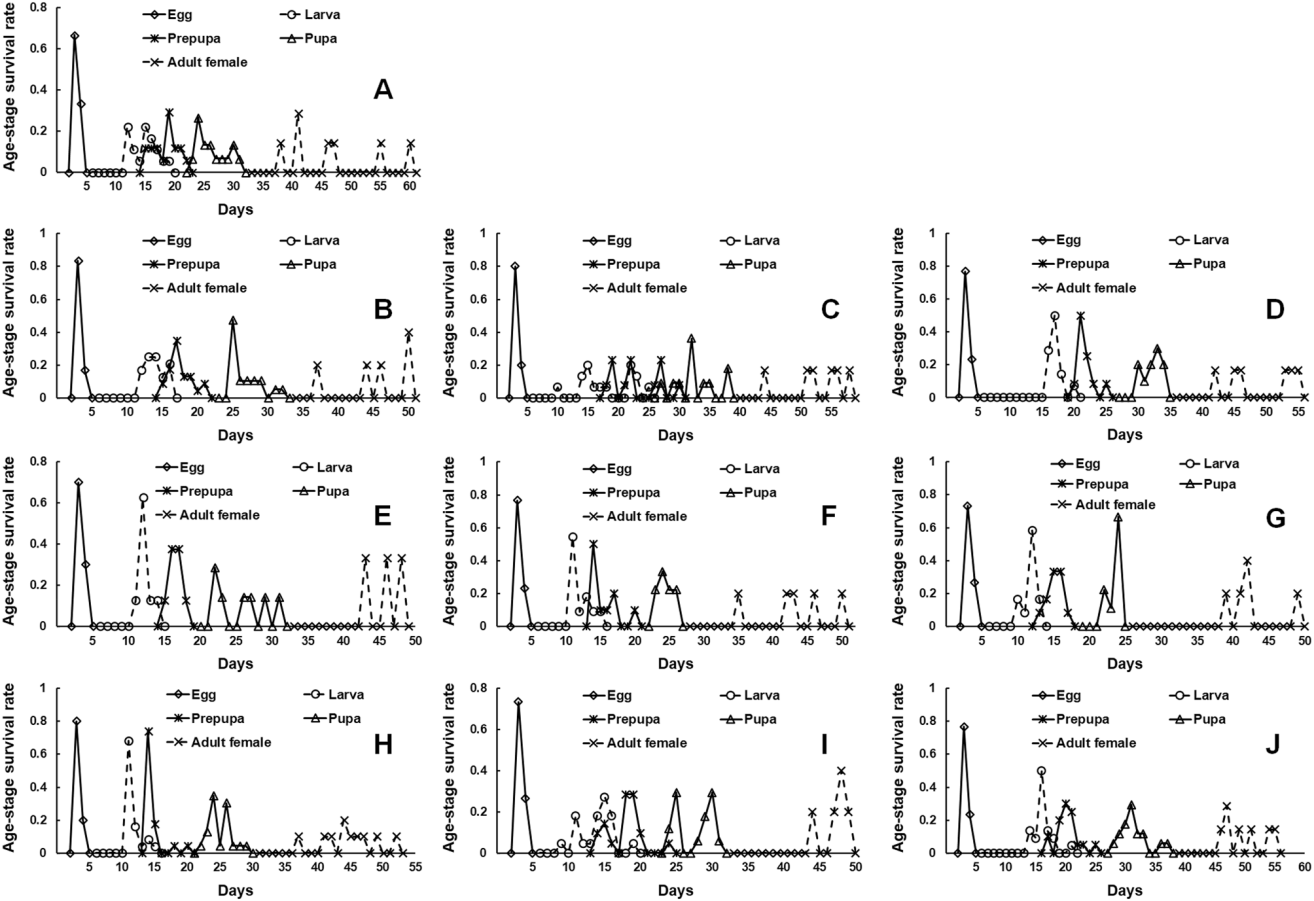
Age-stage survival rate (*S*_*xj*_) of *Grapholita molesta* in plum collected on (**A**) May 11, (**B**) May 31, (**C**) June 11, and (**D**) June 25; peach collected on (**E**) May 31, (**F**) June 11, and (**G**) June 25; and apple collected on (**H**) May 31, (**I**) June 11 and (**J**) June 25.

Age- and stage-specific survival rates of *G. molesta* larvae were highest on 11th day in both peach and apple collected on May 31 (Fig. 1). The peaks of larval survival rate became extended as fruit collection dates in plum and apple. But, the peaks of larval survival rate appeared between 10 and 15th day in peach.

### Longevity and fecundity of adult females

Preoviposition period and fecundity of *G*. *molesta* were significantly affected by fruit species (preoviposition period *F* = 6.84, *df* = 2, 27, *P* = 0.004; fecundity *F* = 30.21, *df* = 2, 27, *P* < 0.001), by collection date (preoviposition period *F* = 3.21, *df* = 2, 27, *P* = 0.065; fecundity *F* = 4.25, *df* = 2, 27, *P* = 0.024), or by fruit species by collection date interaction (preoviposition period *F* = 7.44, *df* = 4, 27, *P* < 0.001; fecundity *F* = 7.45, *df* = 4, 27, *P* < 0.001) (Table 3).

**Table 3:** Fecundity of female of *Grapholita molesta* moths reared as larvae on different fruits under laboratory conditions.

Fruit	Collected on	Preoviposition period (d)	Oviposition period (d)	Fecundity	Longevity (d)
Plum	May 11^ǂ^	3.60 ± 0.24b^§^	18.00 ± 2.43	135.00 ± 16.43b	21.60 ± 2.46
	May 31	3.25 ± 0.25bB^§§^	18.00 ± 1.47	205.75 ± 27.21aAB	21.25 ± 1.44
	June 11	3.60 ± 0.24bA	17.40 ± 0.68	144.00 ± 14.60abC	21.00 ± 0.76
	June 25	4.67 ± 0.33aA	16.33 ± 0.33	150.67 ± 14.81abB	21.00 ± 0.58
Peach	May 31	5.33 ± 0.33aA	13.67 ± 1.45	116.33 ± 15.59cB	19.00 ± 1.15
	June 11	3.25 ± 0.25bA	17.50 ± 1.55	202.00 ± 14.46bB	20.75 ± 1.80
	June 25	3.75 ± 0.48bAB	16.75 ± 1.11	258.25 ± 7.60aA	20.50 ± 0.50
Apple	May 31	3.20 ± 0.20aB	19.20 ± 1.66	273.40 ± 20.70aA	22.40 ± 0.78
	June 11	3.33 ± 0.33aA	16.33 ± 0.33	287.00 ± 19.22aA	19.67 ± 0.33
	June 25	3.20 ± 0.20aB	18.60 ± 1.36	281.80 ± 16.81aA	21.80 ± 1.50

^ǂ^Excluded in two-way ANOVA.

^§^Means followed by different lower case letter are significantly different among collection dates within a fruit species.

^§§^Means followed by different upper case letter are significantly different among host fruits in each collection date.

The preoviposition period within plum fruits was longer on June 25 (4.67 d) compared to those collected earlier. But, preoviposition period was longer on May 31 than later dates in peach while it was similar among all collection dates in apple.

Among fruit species, the highest fecundity was found for moths reared as larvae in peach (258.25) and apple (281.80) collected on June 25. Within each fruit species, fecundity was highest (205.75) in moths reared as larvae in plums collected on May 31, compared to those from fruit collected earlier. Interestingly, in peach fruit, fecundity gradually increased with fruit age and was greatest in fruits collected on June 25. Apple on all dates showed higher fecundity.

However, oviposition period and adult longevity were not affected by fruit species (oviposition period *F* = 1.93, *df* = 2, 27, *P* = 0.164; longevity *F* = 0.67, *df* = 2, 27, *P* = 0.522), by collection date (oviposition period *F* = 0.04, *df* = 2, 27, *P* = 0.964; longevity *F* = 0.18, *df* = 2, 27, *P* = 0.840), or by fruit species by collection date interaction (oviposition period *F* = 1.75, *df* = 4, 27, *P* = 0.167; longevity *F* = 0.61, *df* = 4, 27, *P* = 0.662). Peach fruits collected on June 25 or apple fruits (irrespective of collection date) were the best food sources for *G. molesta* larvae.

### Fruit firmness

**Figure 2:**
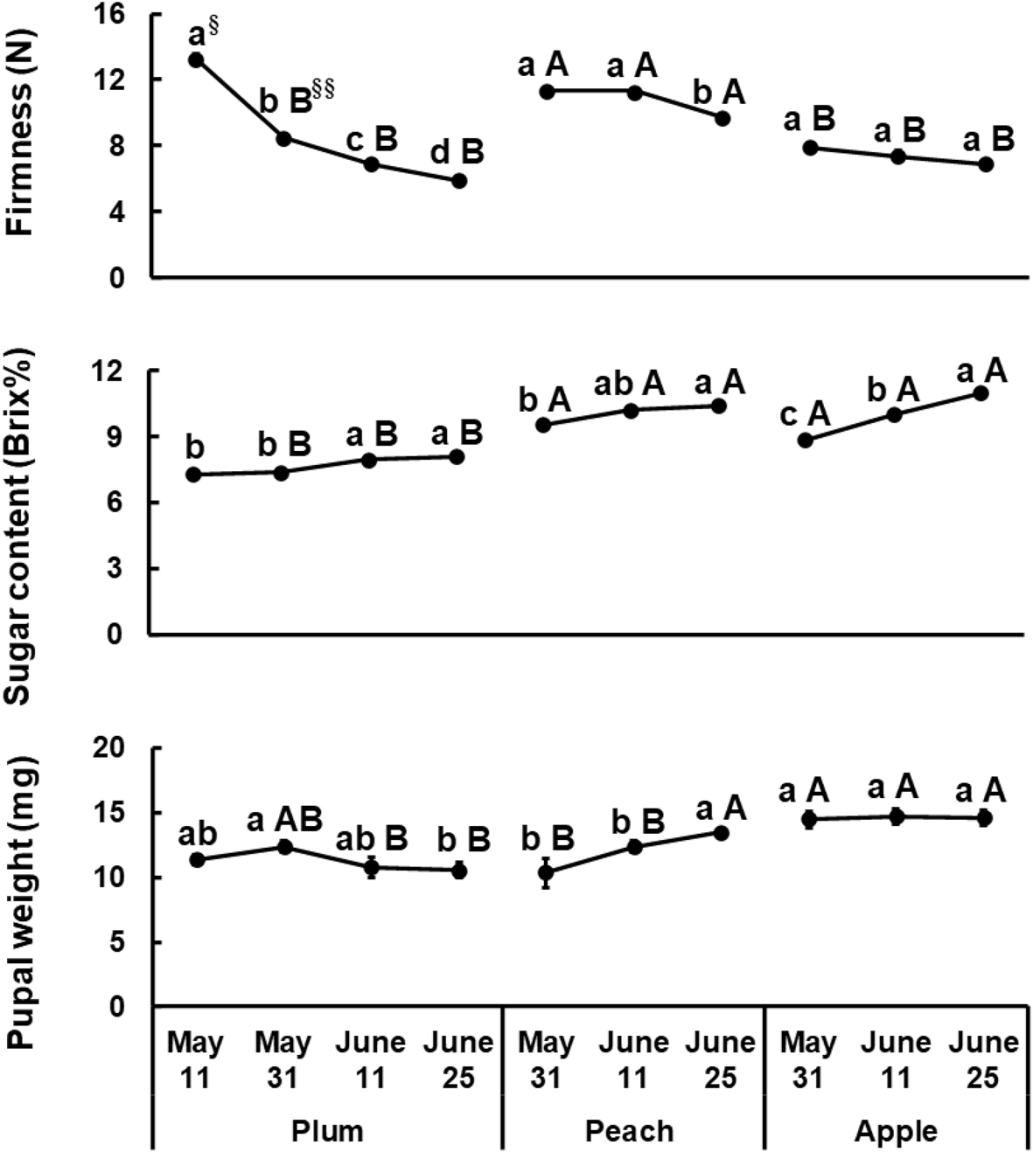
Fruit hardness and sugar content of fruits, compared to pupal weights of *Grapholita molesta* on rearing fruit. Means followed by different lower case (^§^) letter are significantly different among collection dates within a fruit species and different upper case letter (^§§^) are significantly different among host fruits in each collect date.

Firmness of the fruit surface was influenced by fruit species (*F* = 214.60, *df* = 2, 73, *P* < 0.001), by collection date (*F* = 44.01, *df* = 2, 73, *P* < 0.001), or by fruit species by collection date interaction (*F* = 4.61, *df* = 4, 73, *P* = 0.002) (Fig. 2). For all fruits, fruit firmness gradually decreased over time. Plum fruits collected on May 11 showed highest firmness (13.30 N), then firmness decreased quickly as collection date. Among all fruit species, the fruit firmness was greater in peach when collected on May 31 (11.37 N) or June 11 (11.34 N) compared to that for plum or apple.

### Fruit sucrose content

Sugar content was also greatly affected by fruit species (*F* = 211.45, *df* = 2, 78, *P* < 0.001), by collection date (*F* = 60.77, *df* = 2, 78, *P* < 0.001), or by fruit species by collection date interaction (*F* = 6.62, *df* = 4, 78, *P* < 0.001) (Fig. 2). Sugar content in plum and apple increased with collection date. The sugar content in all fruits was highest on June 25 than those collected on May 31. Among all fruit species and stages, sugar content was highest in peach and apple collected on June 25.

### Pupal weights of larvae reared on different fruits

Pupal weights of *G. molesta* were affected by fruit species (*F* = 15.64, *df* = 2, 125, *P* < 0.001), by collection date (*F* = 5.51, *df* = 2, 125, *P* = 0.005), or by fruit by collection date interaction (*F* = 10.57, *df* = 4, 125, *P* < 0.001) (Fig. 2). The highest pupal weight (13.50 mg) was found in peach collected on June 25 and apple for all collection dates. In peach, pupal weight increased with fruit age (= collection date), but did not increase in plum or apple with increased fruit age.

### Effect of firmness and sugar content on larval duration, pupal weight, and fecundity

A significant regression was found between firmness and pupal weight in peach (y = − 1.32x + 26.34; R^2^ = 0.214; *F* = 6.80, *df* = 1, 25, *P* = 0.015), firmness and fecundity in peach (y = − 58.59x + 830.05; R^2^ = 0.576; *F* = 12.25, *df* = 1, 9, *P* = 0.007), firmness and larval duration in plum (y = − 0.37x + 15.73; R^2^ = 0.112; *F* = 8.74, *df* = 1, 69, *P* = 0.004), and firmness and larval duration in apple (y = − 4.42x + 43.18; R^2^ = 0.554; *F* = 83.23, *df* = 1, 67, *P* < 0.001). No relationship was found between firmness and pupal weight or fecundity in both plum and apple or between firmness and larval duration in peach.

Significant regression was found between sugar content and pupal weight in plum (y = − 1.82x + 25.31; R^2^ = 0.078; *F* = 4.91, *df* = 1, 58, *P* = 0.031), sugar content and pupal weight in peach (y = 3.37x − 21.80; R^2^ = 0.335; *F* = 12.61, *df* = 1, 25, *P* = 0.002), sugar content and fecundity in peach (y = 148.52x − 1301.14; R^2^ = 0.844; *F* = 48.72, *df* = 1, 9, *P* < 0.001), sugar content and larval duration in plum (y = 4.66x − 23.08; R^2^ = 0.263; *F* = 24.60, *df* = 1, 69, *P* < 0.001), and sugar content and larval duration in apple (y = 1.99x − 9.21; R^2^ = 0.554; *F* = 83.11, *df* = 1, 67, *P* < 0.001).

### Fruit effect on life history parameters

Significant differences were observed among fruit species and stages in doubling time (*DT*), finite rate of increase (*λ*), intrinsic rate of increase (*r*_*m*_), net reproductive rate (*R*_*O*_), and mean generation time (*T*) (Table 4). Doubling time in plums collected on June 25 was higher than that obtained on May 11 or May 31. In contrast, doubling times of larvae reared on peach fruits were lower on June 25 than May 31. Among fruit species, doubling time was higher in plum than in peach or apple collected on June 25.

**Table 4:** Life table parameters of *Grapholita molesta* reared as larvae on different fruits.

Fruit	Collected on	Parameters (mean ± SE)				
		*DT*	*λ*	*r*_*m*_	${R}_{O}$	*T* (day)
Plum	May 11	5.87 ± 0.27b^§^	1.13 ± 0.01a	0.12 ± 0.01a	31.73 ± 3.86a	29.37 ± 1.16b
	May 31	5.36 ± 0.37bB^§§^	1.14 ± 0.01aB	0.13 ± 0.01aB	38.89 ± 5.14aB	28.46 ± 1.30bA
	June 11	6.96 ± 0.34aB	1.10 ± 0.01bB	0.10 ± 0.00bB	29.30 ± 2.97aB	34.03 ± 1.02aA
	June 25	7.09 ± 0.30aB	1.10 ± 0.00bB	0.10 ± 0.00bB	29.83 ± 2.93aC	34.84 ± 0.62aA
Peach	May 31	8.31 ± 0.38aA	1.09 ± 0.00bC	0.08 ± 0.00bC	10.70 ± 1.43cC	28.53 ± 1.90aA
	June 11	5.22 ± 0.08bA	1.14 ± 0.00aA	0.13 ± 0.00aA	33.94 ± 2.43bB	26.58 ± 0.86aB
	June 25	4.73 ± 0.21bA	1.16 ± 0.01aA	0.15 ± 0.01aA	43.39 ± 1.28aB	25.72 ± 1.13aC
Apple	May 31	4.22 ± 0.14bC	1.18 ± 0.01aA	0.16 ± 0.01aA	90.52 ± 6.85aA	27.44 ± 1.15aA
	June 11	5.20 ± 0.19aA	1.14 ± 0.01bA	0.13 ± 0.00bA	47.44 ± 3.18cA	28.95 ± 1.32aB
	June 25	5.04 ± 0.18aA	1.15 ± 0.01bA	0.14 ± 0.00bA	65.86 ± 3.93bA	30.46 ± 0.82aB

Student’s *t* test was conducted for pairwise group comparison at *P* < 0.05. *DT* doubling time, *λ* finite rate of increase, *r*_*m*_ intrinsic rate of increase, *R*_*O*_ net reproductive rate, *T* mean generation time.

^§^Means followed by different lower case letter are significantly different among collection dates within a fruit species.

^§§^Means followed by different upper case letter are significantly different among host fruits in each collection date.

The finite rate of increase (*λ*) and the intrinsic rate of increase (*r*_*m*_) for larvae reared on plum was lower on fruits collected June 25 than May 11 or May 31. On the other hand, the finite rate of increase (λ) and intrinsic rate of increase (*r*_*m*_) were higher in peach collected on June 25 compared to those fruits collected on May 31. The net reproductive rate (*R*_*O*_) was highest on May 31 than later collection date within apple. Among fruit species, net reproductive rate (*R*_*O*_) was also found highest on May 31 in apple compared to peaches or plums on all collection dates. The mean generation time (*T*) was shortest in peach collected on June 25 compared to plum or apple collected on the same date. So, from the life table parameters perspective, peaches and apples collected on June 25 and May 31, respectively, were the best food sources for *G. molesta*.

## Discussion

The availability of host plants is a significant factor determining the population dynamics of herbivorous insects^1,13,14^. The physical characteristics and chemical components of host plants vary with their phenological stages^16,17,27–29^, and this variation affects the performance of herbivorous insects, including development time, survival rate, and fecundity^13,30^. Stone fruits such as peach (*P. persica*), plum (*P. domestica*), and nectarine (*P. persica* var. nectarina) serve as primary hosts for *G. molesta,* whereas the pome fruits apple (*M. domestica*) and pear (*Pyrus communis* L.) are considered secondary hosts^2,4^. Myers et al.^22^ states that larval survival of *G. molesta* is better on ripening peach fruit than immature apple fruit, but they did not show the compounding effect of fruit species and fruit’s developmental stage. In our previous study where the suitability of immature fruits were examined^26^, immature apple fruits were found to be better for development of *G. molesta* than immature peach fruit. Thus, in this study, we further evaluated the effect of developmental characteristics of three kinds of fruits on the development and fecundity performance of *G. molesta* along with an examination of firmness and sugar content of the fruits.

We found that fruit species and fruit developmental stage both significantly affected the larval development, reproduction, and life table parameters of *G. molesta,* although the duration of the egg stage of *G. molesta* was not affected, findings that are similar to Sarker and Lim^26^ and Du et al.^1^. The duration of the larval stage was shortest in peach fruits on June 11 and June 25 compared to all plum or apple fruits collected on the same dates. Myers et al.^21^ and Najar-Rodriguez et al.^3^ also found that larvae of *G. molesta* developed faster in peach than in apple. The larval-pupal duration was also shorter in peach collected on June than in plum or apple collected in the same period. Similarly, Du et al.^1^ found that the immature period of *G. molesta* was shorter in peach than in pear, which might be caused by the relatively high sugar content of peaches. Nevertheless, fruit quality could directly affect such development of *G. molesta* in various ways including physical condition, nutrition, and phenolic compounds^31,32^. Larval duration increased with fruit maturity (i.e., collection date) in plum and apple, similar to another study by Du et al.^1^ in which larval duration of *G. molesta* became longer in pear fruit collected in the late season compared to in fruits collected the early season.

The fruit penetration rate of first instars was greatly affected by the firmness of the fruits and was lowest in peach with the highest firmness compared to plum and apple, all collected on May 31. However, plum fruits collected on May 11 were firmer than the peach collected on May 31 but showed a higher entering rate compared to the peach. Thus, firmness might not be the only cause for the failure on the part of *G. molesta* to enter fruits. According to previous studies, the long and dense hair of peach fruits make larval penetration more difficult^33,34^. Reduction in pubescence results in higher fecundity for *Cydia pomonella* (L.) in peach fruits^35,36^. Westgard et al.^37^ and Plourde et al.^38^ also demonstrated that pubescence is related with deterrence of *C*. *pomonella*. Therefore, in peach, the fruit penetration rate might be similarly low in all developmental periods regardless of firmness even though reproduction and development was better for those larvae in the fruit. This might be the reason for the relatively low total survival rate found in peach, and similarly, Du et al.^1^ found that preimaginal survival rate of *G. molesta* was 30% in peach fruit collected on June 25. This explanation is supported by the life table analysis where we found the highest *r*_*m*_ value and pupal weight in peach fruit collected on June 25.

The preoviposition period of *G. molesta* females was longest on late-collected plum and early-collected peach. The fecundity and pupal weight were also lowest on late-collected plum and early-collected peach. Similarly, Sarker and Lim^26^ found that the preoviposition period of *G. molesta* in peach was greater on fruits collected on May 25 compared to plum or apple collected at the same time. Du et al.^1^ also found that both parameters, preoviposition period and pupal weight, were poor on late season pear. Late-collected plums may not be a good food source because it liquefies quickly. Poor food quality typically results in lower growth rates, smaller body sizes, and reduced fecundity^39,40^. The preoviposition period may be negatively correlated with pupal weight, as numerous studies reported that pupal weight is positively correlated with fecundity, and both pupal weight and fecundity of lepidopteran pests are influenced by host plants^3,41–44^.

In plum, we found better development, reproduction, and intrinsic rate of increase (*r*_*m*_) in fruit collected early except fruits collected on May 11 which show much higher firmness. However, plum fruits collected late had very low firmness, and thus were not suitable especially for the development of *G. molesta*. In contrast, development, reproduction, and intrinsic rate of increase (*r*_*m*_) were better in the late-collected peach fruit which had a higher firmness and higher sugar content than plum. According to Van Steenwyk et al.^17^, as pear fruits grow, stone cells break down in late June to mid-July, and the fruits become more suitable for infestation and development for *C*. *pomonella*. However, by late July through mid-August, the larvae less likely to complete development because the pears are starting to rot and liquefy faster. This may be a similar reason to why plum fruits collected on June 25 were less suitable even though sugar content was higher. The firmness of apple fruits collected on June 25 was similar to plum collected on June 11, but the sugar content of the apple was higher than the plum. Firmness and sugar content are essential for larval development and reproduction^17,45^. This study will help to establish the firmness and sugar content of fruits required for the development and reproduction of *G. molesta.*

Understanding the relationship between the life history parameters of an insect and developmental periods of host plants improves the accuracy of forecasting population dynamics and contributes to the practical application of integrated pest management programs. Both firmness and sugar content are important factors for the development and reproduction of *G. molesta*. In general, peach was better for the development of *G. molesta* as it has a higher sugar content, but it showed a lower penetration rate and survival rate, irrespective of fruit developmental period. Plum, while showing a higher larval penetration rate, were generally unsuitable for development and reproduction due to its rapid liquefaction. In apple, highest levels of fruit penetration, moth reproduction, and overall *r*_*m*_ were observed, probably due to apple having proper firmness and higher sugar content. Thus, for development and reproduction, apple fruit would be the best food source for *G*. *molesta*. However, further studies are needed to assess the effects of other components such as proteins and lipids of those fruit on the life table parameters of *G. molesta*.

## Methods

### Insect rearing

Larvae of *G*. *molesta* were reared in apples fruit described by Sarker and Lim^46^. Apples infested with oriental fruit moth larvae were collected and kept in a growth chamber (DS-11BPL, Dasol Scientific Co. Ltd, Hwaseong, Republic of Korea) at 24.92 ± 0.11 °C, 50.21 ± 1.33% RH, and a 16:8 h (L:D) photoperiod. When the larvae reached the fifth instar, they emerged from the apples and spun cocoons in the paper towel provided for pupation. Pupae were collected from paper towel and placed in breeding dishes (10.0 D × 4.0 H cm, 310102, SPL, Pocheon, Republic of Korea). After the emergence of adult moths, they were moved into ventilated acrylic cylinders (25.5 H × 8.5 D cm) and provided with a piece of cotton soaked in 10% sugar solution as a food source. The acrylic cylinders were held in a desiccator (36.0 L × 28.0 W × 25.0 H cm) and incubated at 25.63 ± 0.14 °C and 91.22 ± 0.12% RH. Once moths had begun laying eggs on the cylinder walls, cages were changed daily to obtain age-specific cohorts of eggs. In a separate incubator, acrylic cylinders with eggs on the walls were kept before the egg hatch, and the first instar larvae were collected for life table experiments or further mass rearing. For our experiments, we reared *G. molesta* for eight generations, and, to minimize inbreeding depression, wild males were added to the rearing population periodically.

### Plant materials

In this study, three kinds of fruits (plum, peach, and apple) were used. Fruits of plum (*P. domestica* variety “Royal Daeseok), peach [*P. persica* locally collected unknown variety), and apple (*M. domestica* variety “Fuji,” strain ‘Busa’) were collected from Iljeek, Dosan, and Gilan Counties, respectively, near Andong, Republic of Korea, in 2017. Plum fruits were collected at four developmental stages (on May 11, May 31, June 11, and June 25), and peach and apple fruits were collected on three developmental stages (on May 31, June 11, and June 25) in 2017. All the orchards were free of pesticide use. The approximate dates of bloom for plum, peach, and apple are mid March-mid April, early April–mid April, and April, respectively. The dates of fruit setting for plum, peach, and apple are mid April–late April, April–May, and April–May, respectively. The harvest time for plum, peach, and apple are mid June–August, July–October, and late October–early November^47^. The diameter of all fruits used in the life table study were 1.77 ± 0.04, 2.67 ± 0.03, 4.06 ± 0.07, and 4.38 ± 0.05 cm when the plum fruits were collected on May 11, May 31, June 11, and June 25; 2.34 ± 0.03, 3.09 ± 0.10, and 3.71 ± 0.05 cm for peach fruits collected on May 31, June 11, and June 25; and 3.40 ± 0.06, 3.80 ± 0.33, and 4.00 ± 0.04 for apple fruit collected on these same dates, respectively. After washing fruits with warm water, they were sealed in plastic zip-lock bags and held at 4 °C in a refrigerator before being used in the assays within 2 days.

### Stage development and survival

In this experiment, field-collected fruits were placed in a breeding dish (10.0 D × 4.0 H, 310102, SPL, Pocheon, Republic of Korea), and one *G. molesta* larva (< 6 h old) was released on each fruit and held at 24.85 ± 0.10 °C, 43.74 ± 0.61% RH, and a 16:8 h (L:D) photoperiod in the incubator. Thirty larvae were used in each treatment. Once a mature larva emerged from a fruit, it was transferred for pupation into another breeding dish filled with tissue papers. The duration of the larval stage was measured as the time from egg hatch to the exit of the mature larva from the fruit; the duration of the prepupal stage was defined as the period from the exit of the mature larva from the fruit to the pupation; and the duration of the pupal stage was from the pupation to the adult emergence.

### Longevity and fecundity of adult females

When adults emerged, they were transferred to transparent square breeding dishes (7.2 L × 7.2 W × 10.0 H cm SPL, Pocheon, Republic of Korea, each with three 40 mm-mesh screens; one per side) and held with one male and one female moth per dish. To provide a carbohydrate source, a cotton wick soaked in 10% sugar solution was placed in each container. Freshly laid eggs on the surface of the container were marked and recorded daily until the adult female died. To avoid pathogenic infections, breeding dishes were replaced daily with new ones. An absence of spermatophores was used to indicate an unmated female, and these moths were excluded from the analysis^1^.

### Measurement of firmness of fruit surface

Fruit firmness was measured with a texture analyzer (EZTest/ CE-346-51990, Shimadzu Corporation, Nakagyo, Kyoto, Japan), which determined the penetration force needed to press a plunger into the fruit. Firmness is the maximum test force loaded on the fruits before penetration, and readings were in Newtons (N). Firmness was measured for each of ten randomly selected fruits in each developmental stage of each species.

### Measurement of sucrose content in fruit

A refractometer (HI 96801 Refractometer, Hanna Instruments Inc., Woonsocket, Rhode Island), was used to determine sucrose levels of fruits by measuring the refractive index to determine the % Brix of sugar in an aqueous solution (where one degree Brix equals 1 g of sucrose in 100 g of solution). To get aqueous solution, fruit parts (exocarp or mesocarp) were placed in a 2 mL microtube (14217394, Axygen, Corning, NY) and a tissue grinder was used to crush the fruit parts. A plastic pipette was then used to collect a sample of this solution and place it onto the prism surface of the refractometer. Ten randomly selected fruits were used for such measurements at each developmental stage of each species, with three replicates of each type (exocarp and mesocarp). These three samples were collected from different positions on each sample fruit and averaged within fruits.

### Statistical analysis

To analyze the effect of fruit species and fruit stage on egg duration, oviposition period, preoviposition period, fecundity, the firmness of fruit surface, and sucrose content of fruits, two-way ANOVA was conducted by using PROC GLM in SAS^48^ after checking for normality and homogeneity using a Kolmogorov–Smirnov test and a Levene test, respectively. In the case of data with heterogeneity (duration of larva, prepupa, pupa, and larva-pupa stage, longevity, and pupal weight), Friedman's two-way non-parametric ANOVA was conducted using SAS 9.4^48^. Data of plum on May 11 were excluded from the two-way ANOVA analysis. After that, one-way parametric or non-parametric ANOVA (weighted least squares method) was performed for the multiple comparison (Bonferroni *t* test) within fruit species on different collection date and among fruit species on each collection date using SAS 9.4^48^.

Similarly, hatching rates of eggs, fruit penetration rates of first instars, fruit exiting rates of mature larva, larval survival rates, pupation rates, emergence rates, and total immature survival rates were analyzed with Friedman's two-way ANOVA. Data of plum on May 11 were excluded from the analysis. For the multiple comparison within fruit species on different collection date and among fruit species on each collection date, chi-square tests with a post-hoc multiple comparison analogous to Tukey’s test^49^ were used.

Larval duration, pupal weight, and fecundity were regressed against fruit firmness or sugar content using SAS 9.4^48^.

The jackknife procedure was performed to test the differences within fruit species on different collection date and among fruit species on each collection date in population parameters, i.e., doubling time (*DT*), finite rate of increase (*λ*), intrinsic rate of increase (*r*_*m*_), net production rate (*R*_*O*_), and mean generation time of (*T*)^50^. The survival rate (*S*_*xj*_) (*x* = age, *j* = stage) is the probability that a newly laid egg would survive to age *x* and stage *j*. Jackknife algorithms for estimating the means and variances and constructing confidence intervals were described only for *R*_*O*_ (the net contribution of each female to the next generation, expressed as the total female offspring per female over the whole oviposition period)^50^. The same procedures were used to estimate other parameters (*r*_*m*_, *T*, *DT*, and *λ*). All fertility data in the life tables were entered in a computer program (LIFETABLE.SAS)^50^ and analyzed using SAS 9.4^48^.

### Ethics statement

This research work is the authors' own original work, which has not been previously published elsewhere. The paper is not currently being considered for publication elsewhere. The paper reflects the authors' own research and analysis in a truthful and complete manner.
